# Insights into Plant–Soil–Microbe Interactions

**DOI:** 10.3390/microorganisms13061183

**Published:** 2025-05-22

**Authors:** Wajid Zaman

**Affiliations:** Department of Life Sciences, Yeungnam University, Gyeongsan 38541, Republic of Korea; wajidzaman@yu.ac.kr

The interactions between plants, soil, and microorganisms play a pivotal role in shaping ecosystem health, biodiversity, and agricultural productivity [1]. As our understanding of these complex interactions deepens, new opportunities emerge for improving agricultural sustainability, soil health, and plant resilience [2]. This Special Issue presents cutting-edge research that explores the dynamic roles of microorganisms in improving plant resilience, promoting soil health, and enhancing agricultural productivity.

Microbial communities residing in the rhizosphere and soil environment significantly influence plant growth, nutrient uptake, and stress tolerance [3,4]. Microorganisms, including mycorrhizal fungi, nitrogen-fixing bacteria, and plant-growth-promoting rhizobacteria (PGPR), are integral to enhancing soil fertility and plant productivity, as well as to protecting plants from environmental stresses and diseases [5,6]. As we reflect on the contributions of this Special Issue, it is evident that the exploration of plant–soil–microbe interactions holds tremendous potential for transforming agricultural practices, environmental sustainability, and ecosystem health. The collaborative efforts of scientists from various disciplines have significantly advanced our understanding of how soil microorganisms influence plant growth, stress tolerance, and disease resistance.

This Special Issue brings together diverse research that highlights the role of microbial communities in promoting soil fertility, improving plant resilience, and enhancing agricultural productivity (Figure 1). These contributions pave the way for innovative solutions to address the challenges posed by soil degradation, climate change, and the increasing demand for sustainable farming practices. The studies presented here not only offer insights into the current state of research, but also set the stage for the next generation of microbial-based strategies for improving crop health and soil management. With the growing recognition of the vital role microorganisms play in shaping plant–soil interactions, these studies collectively advance our understanding of how microbial diversity can be harnessed to foster more sustainable and resilient agricultural ecosystems. From biocontrol and plant growth promotion to microbial ecology and soil health, the research in this Special Issue underscores the promise of microbial solutions in addressing global agricultural challenges.

In conclusion, ‘Insights into Plant–Soil–Microbe Interactions’ provides a comprehensive overview of the dynamic interactions that govern plant health, soil fertility, and ecosystem sustainability. The research presented in this issue not only advances our understanding of plant–microbe relationships, but also lays the groundwork for innovative strategies to enhance agricultural productivity in an environmentally sustainable manner.

## Figures and Tables

**Figure 1:**
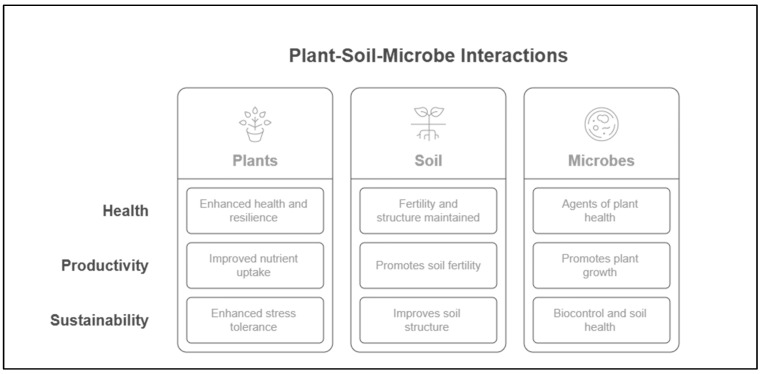
Impact of plant–soil–microbe interactions on health, productivity, and sustainability (created on https://app.napkin.ai. (accessed on 8 April 2025)).

## Data Availability

Not applicable.
